# 

**Published:** 1864-05

**Authors:** 


### CHICAGO MEDICAL JOURNAL.

**Terms, $2.00 per Annum.**

### CONTENTS OF THE MAY NUMBER.

PAGE.

ORIGINAL COMMUNICATIONS.

Alphabet of Auscultation. By Tom O. Edwards, of Lancaster,............ 193
Clinical Lectures on Diseases of the Eye. By E. L. Holmes, M. D., of Chicago, Lecturer on Diseases of the Eye and Ear in Rush Medical College, etc.—The Cornea—Acute Inflammation......... .................. 195
A Case of Perforation of the Intestine. By W. D. Slayter, M. D., of Chicago, Graduate Royal College of Physicians of England, Royal College of Surgeons, &c., London................................................ 199
Diphtheria and its Treatment. By F. R. Mallard, M.D., of Beetown, Wis., 201
A Case for Argument. By M. M. Eaton, Peoria, Ill........................ 206
Inflammation of the Mucous Surface of the Fauces. By G. D. Winch, Asst. Surg. 36th Reg. Wis. Vol.............................................. 207

SELECTED.

On the Treatment of Rheumatic Fever. By Willoughby F. Wade, M. D., M. R. C. P., Senior Physician to the Queen's Hospital, Birmingham, England...................................................................... 208
Trichiniasis in Germany.................................................. 213
Male Fern in Tape Worm................................................... 218
Varicose Veins............................................................ 222
The Gutta Percha Tree...................................................... 223
On a New Mode of Applying Atropine. By Julius Homberger, M. D.... 225

EDITORIAL AND MISCELLANEOUS.............................................. 228

Illinois State Medical Society............................................ 230

BOOK NOTICES AND REVIEWS.................................................. 234

